# Immune responses upon experimental *Erysipelothrix rhusiopathiae* infection of naïve and vaccinated chickens

**DOI:** 10.1186/s13567-020-00830-9

**Published:** 2020-09-14

**Authors:** Eva Wattrang, Helena Eriksson, Tomas Jinnerot, Maria Persson, Elisabeth Bagge, Robert Söderlund, Mohammad Naghizadeh, Tina Sørensen Dalgaard

**Affiliations:** 1 Department of Microbiology, National Veterinary Institute, Uppsala, Sweden; 2 Department of Animal Health and Antimicrobial Strategies, National Veterinary Institute, Uppsala, Sweden; 3 Department of Animal Science, Aarhus University, Tjele, Denmark

**Keywords:** *Erysipelothrix rhusiopathiae*, chicken, leukocyte counts, mannose-binding lectin, IgY, CD45, chicken mannose receptor MRC1L-B

## Abstract

Erysipelas, a disease caused by *Erysipelothrix rhusiopathiae* (ER), is an increasing problem in laying hens housed in cage-free systems. This study aimed to monitor immune responses during ER infection of naïve chickens and chickens vaccinated intra muscularly with a commercial inactivated ER vaccine. Chickens were infected intra muscularly with ER at 30 days of age and blood leukocyte counts, serum levels of mannose binding lectin (MBL) and ER-specific IgY were monitored until the experiment was terminated at day 15 after infection. ER was detected in blood from more chickens and at higher bacterial counts in the naïve group (day 1: 1 of 7 chickens; day 3: 6 of 6 chickens) than in the vaccinated group (day 1: 0 of 7 chickens; day 3: 1 of 6 chickens). During the acute phase of infection transient increases in circulating heterophil numbers and serum MBL levels were detected in all ER infected chickens but these responses were prolonged in chickens from the naïve group compared to vaccinated chickens. Before infection IgY titers to ER in vaccinated chickens did not differ significantly from those of naïve chickens but vaccinated chickens showed significantly increased IgY titers to ER earlier after infection compared to chickens in the naïve group. In conclusion, the ER infection elicited prompt acute innate responses in all chickens. Vaccinated chickens did not have high IgY titers to ER prior to infection but did however show lower levels of bacteraemia and their acute immune responses were of shorter duration.

## Introduction

Poultry products, including eggs, are increasingly important protein food sources worldwide [1, 2] and egg production is forecasted to double globally between 2015 and 2035 [3]. Moreover, with a strong market demand for animal friendly housing systems, egg production is trending to cage free systems including free-range and organic production with access to the outdoors. However, the change in housing systems may also pose a challenge to animal health management with the emergence of new and/re-emergence of old infectious diseases. In modern egg production, the disease erysipelas caused by the bacterium *Erysipelothrix rhusiopathiae* (ER) is one such emerging infectious disease that is associated with cage-free housing and outdoor access [4–10]. The disease manifests as outbreaks with high mortality, up to 60%, and egg production losses. Affected laying hens display acute septicaemia and macroscopic necropsy findings include signs of septicaemia such as splenomegaly, hepatomegaly, petechial hemorrhages on internal organs and occasional valvular endocarditis [4, 6, 7, 11]. Upon histological examination of tissues vascular congestion, intravascular bacterial aggregates, thrombi, necrotic hepatitis and splenitis may be observed. Diagnosis is made by pathologic findings in combination with isolation of ER from liver or spleen [9, 10].

*Erysipelothrix rhusiopathiae* is a Gram-positive facultative anaerobic rod that may infect a wide range of hosts including many mammalian and avian species with or without causing clinical disease [12]. The disease caused by ER has been known since the late nineteenth century but many aspects of this infectious agent still remain unclear. This includes basic knowledge on immune responses and development of protective immunity to ER. In pigs and turkeys vaccination is commonly practiced and generally considered protective against disease [13]. However, failure of vaccines to prevent for example chronic disease has also been reported [13]. Studies in mice have pointed at the importance of phagocytosis by neutrophils and macrophages and the role of specific antibodies in enhancing phagocytosis and subsequent killing of ER [14]. Nonetheless, information on chicken immune responses to ER infection is lacking. In Sweden laying hen flocks affected by an erysipelas outbreak are usually culled for animal welfare reasons. Moreover, on previously affected farms pullets are vaccinated at placement with a single dose of an inactivated vaccine against erysipelas licensed for turkeys as prophylactic measure to prevent further outbreaks [10]. This vaccination strategy is solely based on clinical experience and seems to be effective in many cases although outbreaks in vaccinated flocks have also been reported [10]. The few experimental infection studies of ER in chickens reported in the literature [4, 15–18] have in general focused on the pathogenicity of different ER strains and recovery of bacteria from blood and other organs after infection. One study has also put forward evidence of disseminated intravascular coagulation as part of the pathogenesis [17] and two studies have shown production of ER specific antibodies after experimental infection [15, 16]. Hence, in order to gain more understanding of the chicken immune responses to erysipelas we intended to monitor some basic innate (white blood cell counts and mannose binding lectin; MBL), and specific (IgY titers to ER) immune parameters during infection of naïve and vaccinated chickens. Results would then be able to provide a basis for further studies into the more exact nature of immune mechanisms involved. The present study therefore aimed to set up an ER infection model in chickens without high mortality allowing us to follow these immune parameters during infection. The sampling regime focused on the early responses to infection with frequent sample collection during the week after infection. The chickens were subsequently monitored until day 15 after infection to allow development of IgY to ER. The ER strain used for infection was isolated from a field outbreak of erysipelas in a laying hen flock and is representative of current outbreaks in Swedish laying hen flocks. This strain contains all putative virulence factors as identified by bioinformatic analysis (unpublished data).

## Materials and methods

### Chickens and experimental design

The experiment comprised 39 female Dekalb White Leghorn-type layer hybrids purchased from a commercial hatchery and reared from day-old under SPF-conditions at the animal facilities at the National Veterinary Institute, Uppsala, Sweden. The parent hens were not vaccinated against erysipelas. Chickens were group housed with unlimited access to feed and water in pens on the floor in rooms under negative pressure ventilation. After ER infection uninfected and infected chickens were kept in separate rooms.

All chickens were individually identified by leg rings and at 12 days of age (experimental day −18, days before the experimental infection are indicated as a negative value) chickens were weighed and allocated to three groups to achieve an equal mean weight: group “uninfected”; group “naïve”; and group “vaccinated” (n = 13/group). Within each group chickens were allocated to two sampling groups (n = 7 and 6/group) for alternate sampling after the experimental infection outlined in Additional file 1. At 17 days of age, experimental day −13, chickens in the “vaccinated” group were injected with 0.5 mL of a commercial inactivated erysipelas vaccine containing the ER strain M2 of serotype 2, belonging to clade 2, (Porcilis ERY Vet, MSD Animal Health) in the breast muscle. At 30 days of age, experimental day 0, chickens in the “uninfected” group were injected with 0.5 mL sterile broth and chickens in the “naïve” and “vaccinated” groups were injected with 0.5 mL broth with 10^10^ cfu ER/mL in the breast muscle.

All chickens were monitored daily for clinical signs of disease (e.g. decreased appetite, depression, weakness, ruffled feathers, drooping wings) during the whole experiment and additionally twice daily during the week after infection. All chickens were weighed on experimental days −13, −3, 1 to 5, 8 to 12 and 15. Blood samples were collected from all chickens on experimental days −13, −3 and 15. On experimental days 1, 3, 5, 8 and 11 sampling was only performed in one of the sampling groups of each experimental group (Additional file 2) and thereby individual chickens were only sampled at every other occasion to limit the impact of repeated blood sampling. Approximately 0.5 mL blood was drawn by needle and syringe under sterile conditions from the jugular vein of each chicken on the indicated days. Approximately 350 μL blood was transferred to sterile blood collection tubes with 1.0 mg EDTA K_2_ as additive (#363706, BD Microtainer ^®^ MAP) and the remaining blood was added to sterile test tubes without additives. On experimental day 15 all chickens were killed by cervical dislocation and subjected to post mortem examination.

### Culture of ER inoculate

The ER strain 15-ALD003475, derived from an outbreak of erysipelas in a Swedish laying hen flock in 2015 was used for infection of chickens. Whole-genome SNP comparison with isolates from the study by Forde et al. [19] did not reveal a clear clade assignment for this strain, which could be considered to be of an “intermediate” lineage (unpublished data). The strain was stored at − 70 °C and before preparation of inoculate the strain was maintained in culture on horse blood agar (#B341180; National Veterinary Institute, Uppsala, Sweden). For the inoculate bacteria from an 24 h culture on horse blood agar were cultured for 24 h at 37 °C on a shaker in tryptic soy broth (#B321730, National Veterinary Institute) supplemented with 0.1% Tween 80, 0.1% d-glucose and 20 mg/L l-tryptophan. Numbers of ER in the inoculate was determined by a tenfold serial dilution; 100 µL volumes of each dilution were spread on agar plates, cultured for 48 h at 37 °C, ER colonies were counted and cfu per mL was calculated.

### Re-isolation of ER in samples from infected chickens

In EDTA-stabilised blood samples growth of ER colonies was quantified by direct culture and ER DNA was detected and quantified by PCR assays as previously described [20]. At *post mortem* examination sterile spleen samples were collected and placed in selective sodium-azide crystal-violet broth (# B321051/5, National Veterinary Institute; containing 5 μg/mL crystal-violet and 0.2 mg/mL sodium-azide) and incubated for ER enrichment for 48 h at 37 °C. 10 μL of broth was then spread on horse blood agar plates and incubated for 48 h at 37 °C. The identity of any suspected ER colonies was subsequently verified by matrix-assisted laser desorption/ionization–time-of-flight mass spectrometry (MALDI-TOF MS) on a Biotyper instrument (Bruker).

### Blood leukocyte counts

Absolute counts of heterophilic granulocytes, monocytes, lymphocytes and thrombocytes in EDTA-stabilised whole blood samples were determined using a no-lyse, no-wash flow cytometry based method adapted from the protocol previously described by Seliger et al. [21]. 25 µL of EDTA-stabilised blood was diluted 25-fold in FACS-buffer, i.e. phosphate buffered saline (PBS) supplemented with 0.2% bovine serum albumin (BSA; Sigma-Aldrich), 0.2% sodium azide and 0.05% normal horse serum (Sigma-Aldrich). 50 µL of the diluted blood was subsequently mixed 1:1 with a panel of fluorochrome conjugated antibodies (Table 1) diluted in FACS-buffer and incubated at room temperature for 20 min in the dark where after samples were fixed by addition of 300 µL of FACS-buffer supplemented with paraformaldehyde (PFA; #43368, Alfa Aesar, Thermo Scientific) to a final concentration of 1% PFA. Immediately prior to flow cytometry analysis a fixed volume of fluorescent counting beads (123 count eBeads, #01-1234-42, Invitrogen, Thermo Scientific) was added to each sample. Reverse pipetting was used throughout to pipette precise volumes. Flow cytometry data were recorded for 1 min at reduced flow rate and the gating strategy to define different leukocyte populations is shown in Additional file 2. The number of events counted in the bead gate was according to the manufacturers recommendations at least 1000 and was used to determine the volume of blood sample analysed and calculate absolute numbers of the leukocyte populations. Flow cytometry was performed using a BD FACSCanto™ (BD Biosciences), equipped with 488 nm blue and 633 nm red lasers and results were analysed using the FACSDiva (BD Biosciences) software. Single-stained compensation controls and fluorescence minus one (FMO) negative controls were included in the assays. Titrations of all antibodies were performed to determine optimal labelling conditions prior to the experiment.

**Table 1:** Monoclonal antibodies used for immunolabelling of whole blood for leukocyte counts

Abbreviation	Clone	Specificity	Fluorochrome
KUL01-RPE	KUL01^a^	Chicken mannose receptor MRC1L-B [44]	R-phycoerythrin^c^
CD41/61-Fitc	11C3^b^	Chicken CD41/61 intergrin (GPIIb-IIIa)	Fluorescein^c^
CD45-PerCp/Cy5.5	UM16-6^b^	Chicken CD45, all isoforms [41]	Peridinin chlorophyll-cyanine 5.5^d^

^a^Purchased from Southern Biotech.

^b^Purchased from Bio-Rad Antibodies.

^c^Fluorochrome conjugated by manufacturer.

^d^Fluorochrome conjugated using Lightning-Link™ conjugation kits (Expedeon) according to the manufacturer’s protocol.

### ELISA for detection of chicken mannose binding lectim (MBL)

The MBL serum concentration was measured using an earlier described in house ELISA based on the anti-chicken cMBL antibody HYB182-01 from BioPorto A/S [22, 23].

### ELISA for detection of IgY antibodies to ER

An in house ELISA for detection of antibodies to ER in chicken serum was set up based on an earlier described protocol [15]. For production of coating antigen ER was cultured for 48 h on horse blood agar and colonies were subsequently suspended in 0.05 M Na_2_CO_3_/NaHCO_3_ buffer, pH 9.6, and sonicated at 1.2 A, 10 µm amplitude, for 5 min on ice. Particulate matter was removed by centrifugation at 12 000 × *g* for 20 min, the protein concentration was determined in the supernatant by Bradford assay and the antigen was stored at −20 °C until use. In this study coating antigens prepared from the challenge ER strain 15-ALD003475 or strain 13-ALD025893, which belonged to clade 2 and was more closely related to the vaccine strain according to whole genome sequence SNP analysis (unpublished data) were used respectively. The coating antigen was used at a protein concentration of 5 µg/mL in 0.15 M Na_2_CO_3_/0.35 M NaHCO_3_, pH 9.6, coating buffer, in flat-bottomed 96-well plates (MaxiSorp, Nunc™, ThermoFisher Scientific). PBS with 0.6% BSA was used for blocking and as diluent while PBS with 0.1% BSA was used as wash buffer. Chicken sera were titrated in twofold steps starting at dilutions 1:100 or 1:1000 depending on antibody concentration, to achieve a dilution curve. A high titer serum sample and a negative serum sample were included on each plate as positive and negative controls for plate-to-plate variation. Horseradish peroxidase conjugated polyclonal goat anti chicken IgG (IgY)-Fc antibodies (#AAI29P, BioRad Antibodies) were used as tracer and a commercial substrate buffer (1-Step™ Turbo TMB-ELISA, ThermoFisher Scientific) was used for visualisation of antibody binding. This reaction was stopped at a standardised time point with 1 M H_2_SO_4_ and the A_450_ − A_650_ was measured in an ELISA reader. For each sample the A_450_ − A_650_ values were plotted against the sample dilution and the equation for the linear part of the curve was determined by regression analysis. Antibody titers were then calculated as the dilution that would achieve an A_450_ − A_650_ value of 1.

### Data presentation

Data were presented as group mean values ± 95% confidence intervals (CI) and mean values with non-overlapping CI were treated as rejecting the null hypothesis of no difference. For antibody titers geometrical mean values were calculated, for all other data arithmetic mean values were used. Geometric mean values and CI for geometric mean values were calculated using the software package R 3.5.0.

## Results

### Clinical signs, *post mortem* examination and re-isolation of ER from infected chickens

One chicken in the “naïve” group showed moderate depression on days 2 to 4 after infection and did not gain weight during these days. No other chicken showed any clinical signs of disease during the experiment and the mean weights did not differ significantly between groups during the experiment (group mean weights ± 95% CI, n = 13, for day −3: uninfected 233 ± 13 g; “naïve” 237 ± 14 g; “vaccinated” 235 ± 8 g; for day 15: uninfected 442 ± 19 g; “naïve” 427 ± 20 g; “vaccinated” 442 ± 13 g). Results on detection of live ER by direct culture of blood and detection of bacterial DNA in blood by PCR assays from this experimental infection have been described in detail in [20] (infection trial 3). In summary, on day 1 after infection one of seven sampled chickens and on day 3 after infection all six sampled chickens in the “naïve” group were positive for ER in blood by culture. For the “vaccinated” group, one of six sampled chickens on day 3 after infection was positive for ER in blood by culture.

At *post mortem* examination on day 15 after infection none of the chickens in any of the experimental groups displayed any macroscopic lesions and growth of ER was not detected in any of the spleen samples.

Thus, the results show that ER infection was established in all of the naïve chickens albeit only one of them displayed clinical signs of disease. Vaccination reduced, or possibly protected from, establishment of the infection. Moreover, all of the infected chickens had cleared the infection to an extent where ER was not detectable by culture of blood or spleen or by PCR of blood by the end of the experimental period.

### Blood leukocyte counts during ER infection

**Figure 1:**
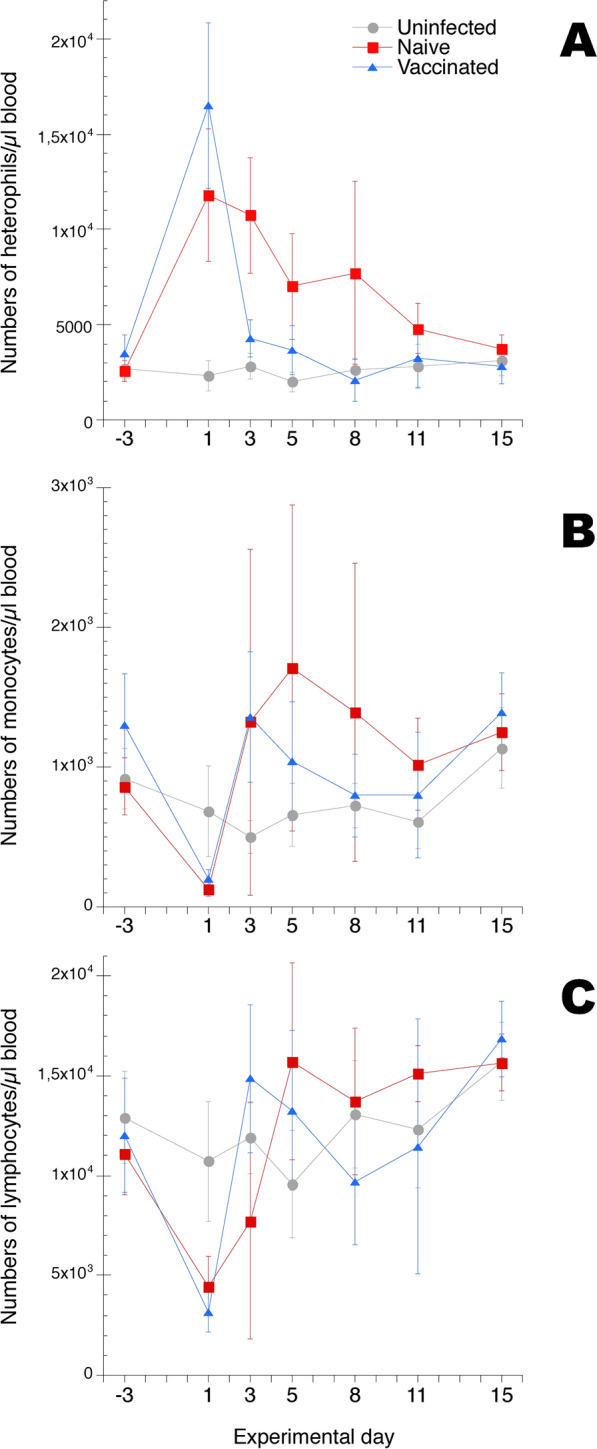
**Total numbers of A heterophils, B monocytes and C lymphocytes in blood.** Chickens were uninfected or experimentally infected with ER on day 0, “naïve” and “vaccinated” groups. Blood samples were collected at the indicated days. Results are shown as group mean values ± 95% CI where non-overlapping CI indicate statistically significant differences. On days −3 and 15 n = 13/group, on days 1, 5 and 8 n = 7/group and on days 3 and 11 n = 6/group. For details see "Materials and methods" section.

Numbers of circulating heterophils (defined by FSC/SSC characteristics and CD45 high expression), monocytes (defined by FSC/SSC characteristics and CD45 high and MRC1L-B expression), lymphocytes (defined by FSC/SSC characteristics and CD45 high expression and as CD41/61 negative) and thrombocytes (defined by CD41/61 expression) in peripheral blood were monitored using a no-lyse, no-wash flow cytometry based method (Additional file 2) at sampling prior to infection, day −3, and during 2 weeks after ER infection (Figure 1). Results showed an approx. sixfold increase in the numbers of circulating heterophils in blood from chickens in both the “naïve” group and the “vaccinated” group on day 1 after ER infection compared to the uninfected chickens (Figure 1A). For the naïve chickens heterophil numbers remained significantly elevated on day 3 after infection and then progressively returned to pre-infection level by the end of the experimental period. For the vaccinated chickens heterophil numbers returned to pre-infection level on day 3 after infection and remained at this level throughout the rest of the experimental period. Numbers of circulating monocytes were significantly decreased in blood from chickens in both the “naïve” group and the “vaccinated” group on day 1 after ER infection compared to the uninfected chickens (Figure 1B). For both groups of ER infected chickens monocyte numbers subsequently increased on day 3 after infection and remained elevated on average, days 3–8 for naïve chickens and days 3–5 for vaccinated chickens respectively, compared to the uninfected chickens. However, on these sampling occasions monocyte numbers for ER infected chickens showed a large variation between individuals and the group mean values were hence not statistically significantly different from those of uninfected control chickens. The total numbers of lymphocytes were also significantly decreased in blood from chickens in both the “naïve” group and the “vaccinated” group on day 1 after ER infection compared to the uninfected chickens (Figure 1C). For both groups of ER infected chickens lymphocyte numbers subsequently increased on day 3 and did not differ significantly from those of uninfected chickens for the remaining experimental period. Numbers of circulating thrombocytes were not significantly influenced by ER infection (Additional file 3).

Thus, ER infection induced a prompt transient heterophilaemia, monocytopenia and lymphopenia in all infected chickens. The heterophilaemia was shorter in duration for the vaccinated chickens compared to the naïve chickens.

### Expression of CD45, chicken mannose receptor MRC1L-B and CD41/61 on circulating leukocyte populations during ER infection

**Figure 2:**
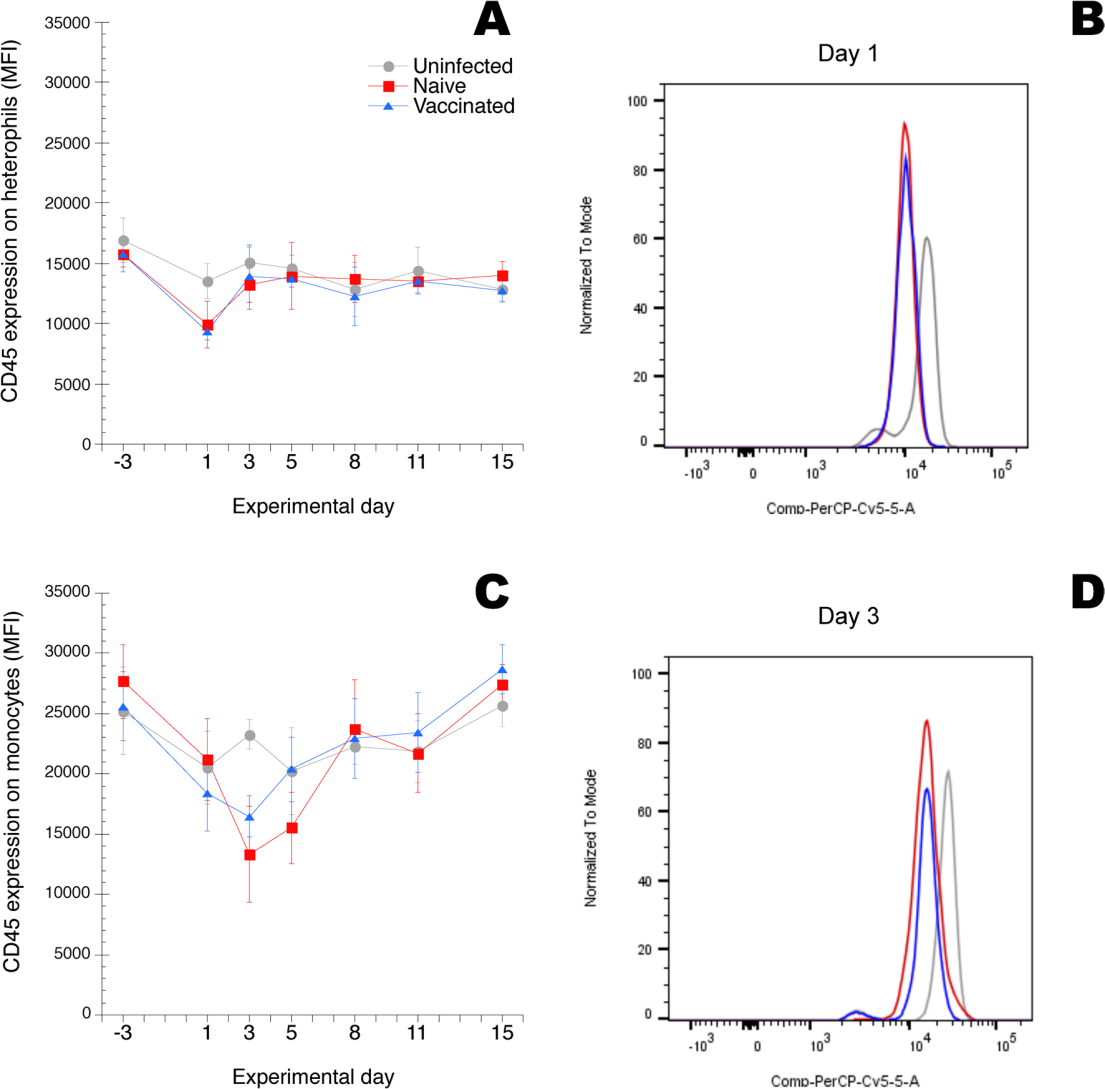
**CD45 expression on A and B heterophils and C and D monocytes.** Chickens were uninfected, or experimentally infected with ER on day 0, “naïve” and “vaccinated” groups. Blood samples were collected at the indicated days. In **A** and **C** expression is shown as median fluorescence intensity (MFI) in the heterophil and monocyte gates, respectively, as defined in Additional file 2, and results are shown as group mean values ± 95% CI where non-overlapping CI indicate statistically significant differences. On days –3 and 15 n = 13/group, on days 1, 5 and 8 n = 7/group and on days 3 and 11 n = 6/group. In **B** CD45 expression on heterophils on day 1 after ER infection is shown as fluorescence intensity for one representative uninfected, “naïve” and “vaccinated” chicken, respectively. In **D** CD45 expression on monocytes on day 3 after ER infection is shown as fluorescence intensity for one representative uninfected, “naïve” and “vaccinated” chicken, respectively. Histograms in **B** and **D** were compiled using the FlowJo™ software package. For details see "Materials and methods" section.

**Figure 3:**
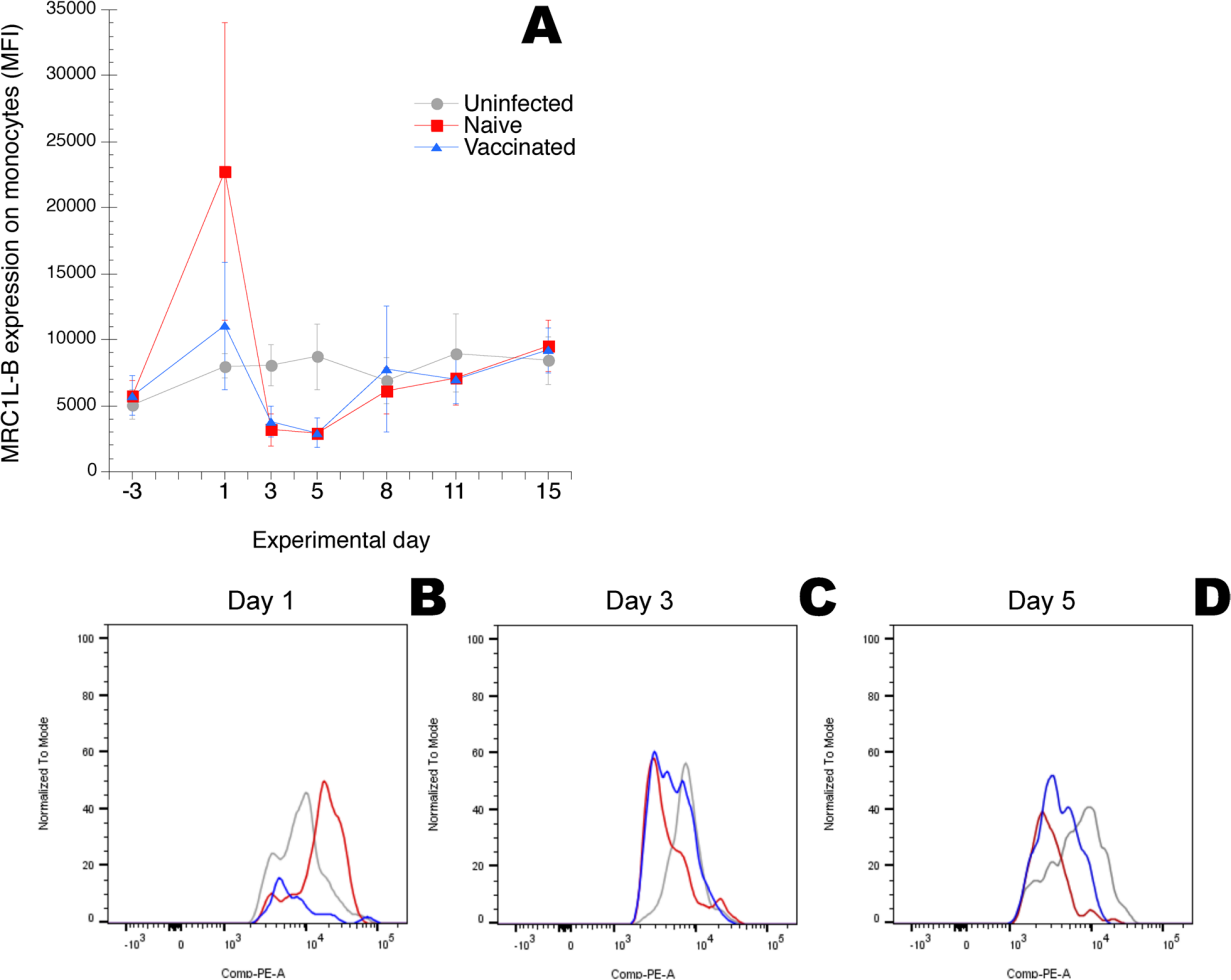
**MRC1L-B expression on monocytes.** Chickens were uninfected, or experimentally infected with ER on day 0, “naïve” and “vaccinated” groups. Blood samples were collected at the indicated days. In **A** expression is shown as median fluorescence intensity (MFI) in the monocyte gate as defined in Additional file 2, and results are shown as group mean values ± 95% CI where non-overlapping CI indicate statistically significant differences. On days –3 and 15 n = 13/group, on days 1, 5 and 8 n = 7/group and on days 3 and 11 n = 6/group. In **B**–**D** MRC1L-B expression on monocytes on **B** day 1, **C** day 3 and **D** day 5 after ER infection is shown as fluorescence intensity for one representative uninfected, “naïve” and “vaccinated” chicken, respectively. Histograms were compiled using the FlowJo™ software package. For details see “Materials and methods” section.

The levels of cell surface expression of CD45 on heterophils, monocytes, lymphocytes and thrombocytes, respectively, of MRC1L-B on monocytes and CD41/61 on thrombocytes detected by the fluorochrome conjugated antibodies described in Table 1 was monitored as median fluorescence intensity (MFI) in the respective leukocyte gates (Additional file 2). Results showed that the CD45 expression was in general higher on monocytes from uninfected chickens compared to that on heterophils from uninfected chickens (Figure 2). For heterophils the CD45 expression was significantly decreased on cells from chickens in both the “naïve” group and the “vaccinated” group on day 1 after ER infection compared to the uninfected chickens (Figures 2A and B). For monocytes the CD45 expression was significantly decreased on cells from chickens in both the “naïve” group and the “vaccinated” group on day 3 after infection compared to the uninfected chickens (Figures 2C and D). For lymphocytes and thrombocytes no significant alterations in the CD45 expression were recorded during the experiment (not shown in figure). On day 1 after ER infection the MRC1L-B expression on monocytes was approx. fivefold increased on cells from chickens in the naïve group compared that on monocytes from uninfected chickens (Figure 3). Subsequently on days 3 and 5 the MRC1L-B expression on monocytes was significantly decreased on cells from both the “naïve” group and the “vaccinated” group compared to that on cells from uninfected chickens. For the CD41/61 expression on thrombocytes no significant alterations were recorded during the experiment (not shown in figure).

Thus, both circulating heterophils and monocytes showed potential signs of activation, monitored as alterations in the cell surface expression of CD45 (heterophils and monocytes) and MRC1L-B (monocytes) during the early phase of ER infection.

### Systemic MBL responses during ER infection

**Figure 4:**
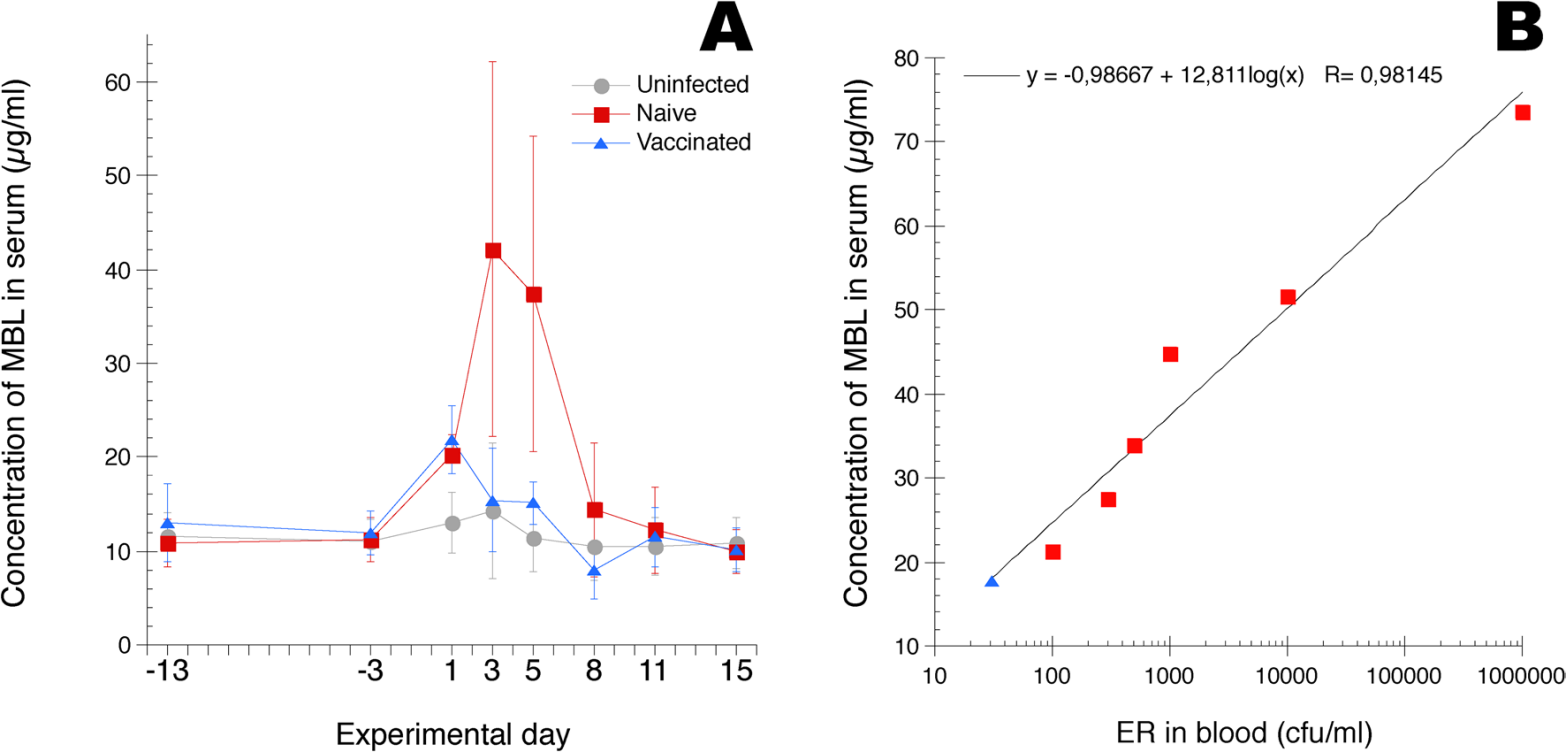
**A MBL concentrations in serum and B correlation between MBL concentrations and ER in blood.** Chickens were uninfected or experimentally infected with ER on day 0, “naïve” and “vaccinated” groups. Sera were collected at the indicated days. Results are shown as group mean values ± 95% CI where non-overlapping CI indicate statistically significant differences. On days −13, −3 and 15 n = 13/group, on days 1, 5 and 8 n = 7/group and on days 3 and 11 n = 6/group. **B** Correlation between MBL concentrations in serum and growth of ER in blood from ER infected chickens on day 3 after infection, “naïve” and “vaccinated” groups. The trend line was calculated using the curve fit logarithmic mode in the software KaleidaGraph, version 4.1.0 (Synergy Software) and equation and R-value are shown in the figure. Results on bacterial counts have previously been presented in [20]. For details see “Materials and methods” section.

Serum MBL levels were monitored at sampling occasions day −13 and day −3 before ER infection and for 2 weeks after the infection (Figure 4A). Pre-infection values were in mean approx. 10 µg MBL/mL serum for all three groups of chickens. On day 1 after ER infection the MBL levels were significantly increased and approx. doubled for both groups of ER infected chickens compared to those of uninfected control chickens. For the naïve chickens serum MBL levels were further significantly increased, approx. fourfold, on day 3 and 5 after infection and subsequently returned to pre-infection levels on day 8 and onward. For the vaccinated chickens, serum MBL returned to pre-infection levels on day 3 and remained at this level throughout the experimental period. On day 3 after ER infection MBL levels in culture positive [20] chickens in the “naïve” group (n = 6) and the “vaccinated” group (n = 1) correlated well to the numbers of bacteria detected in blood (Figure 4B). On day 5 after infection there were no culture positive chickens but three chickens in “naïve” group were positive for ER DNA [20] and these showed the highest MBL values (74.8, 43.0 and 34.4 µg MBL/mL) compared to those of the DNA negative chickens (34.0, 30.8, 26.5 and 18.0 µg MBL/mL) of that group. However, the sole culture positive chicken in the “vaccinated” group on day 3 did not have the highest MBL value of that group and the culture positive and ER DNA positive chicken in the “naïve” group on day 1 after infection did not have the highest MBL value of that group. Similarly, the ER DNA positive chicken in the “vaccinated” group on day 5 did not have the highest MBL value of that group.

Thus, the ER infection elicited a prompt serum MBL response in all infected chickens and this response was more pronounced and prolonged for the naïve chickens compared to the vaccinated chickens. A correlation between ER bacterial load and MBL responses was also indicated in chickens with high bacteraemia.

### Systemic ER-specific IgY responses

Serum IgY titers to the ER challenge strain were monitored at sampling occasions day −13 (i.e. the day of vaccination of chickens in the “vaccinated” group) and day −3 before infection (i.e. 10 days after vaccination) and during 2 weeks after ER infection for the two ER infected groups of chickens, “naïve” and “vaccinated”. For uninfected chickens serum IgY titers to the ER challenge strain were determined on sampling occasions day −13, −3 and 15. In addition, serum IgY titers to the ER “vaccine related” strain were determined on sampling occasions day −13 and 15 for all chicken groups.

**Figure 5:**
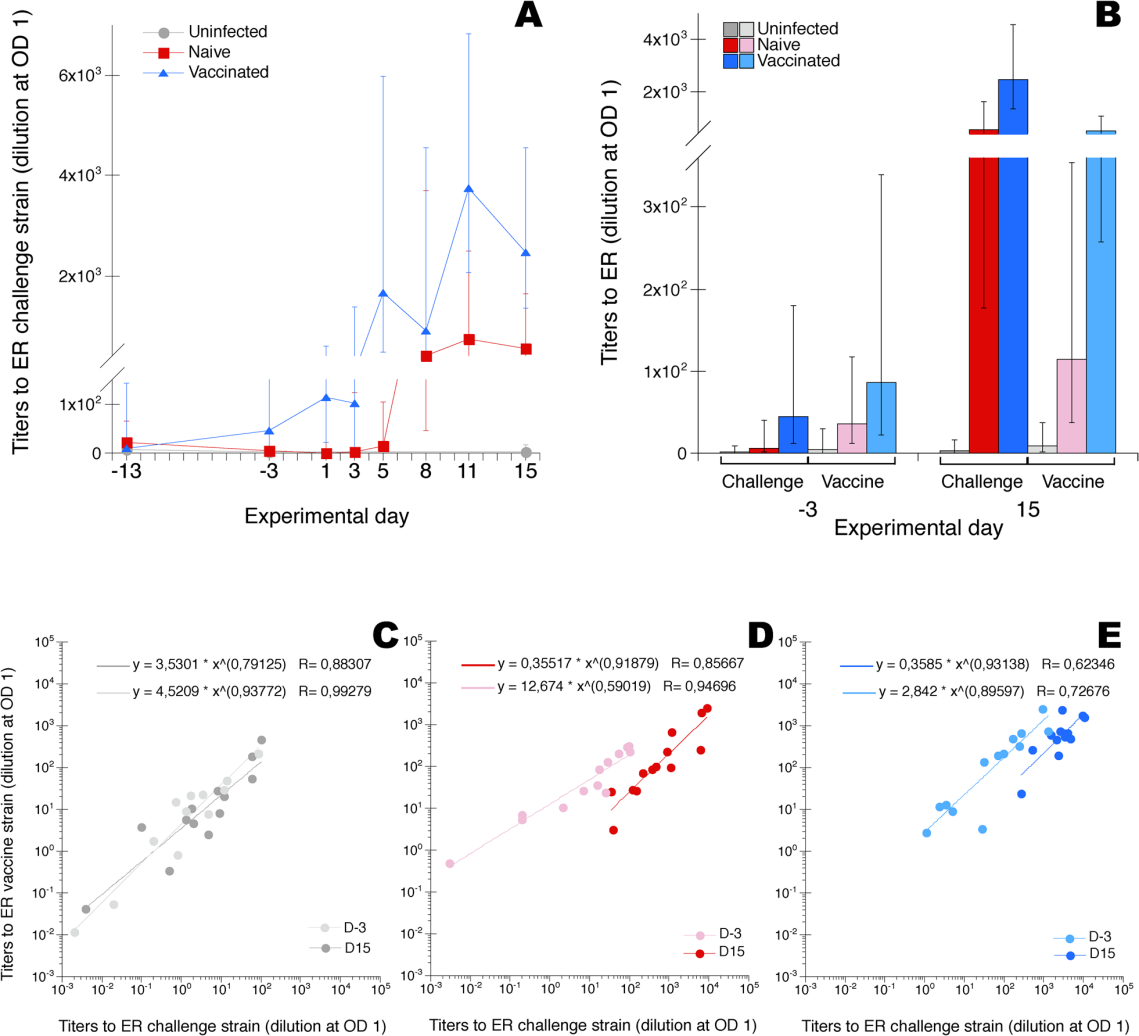
**IgY titers to ER in serum. A** IgY-titers to the ER challenge strain in serum from uninfected chickens or chickens experimentally infected with ER on day 0, “naïve” and “vaccinated” groups. Sera were collected at the indicated days from uninfected chickens, naïve chickens and vaccinated chickens. **B** IgY-titers to the ER challenge strain (Challenge) and the ER “vaccine related” strain (Vaccine), respectively, in sera collected on days −3 and 15, respectively, from uninfected chickens, naïve chickens and vaccinated chickens. **A** and **B** Results are shown as group mean values ± 95% CI where non-overlapping CI indicate statistically significant differences. On days −13, −3 and 15 n = 13/group, on days 1, 5 and 8 n = 7/group and on days 3 and 11 n = 6/group. **C**–**E** IgY-titers to the ER challenge strain (x-axis) and to the ER “vaccine related” strain (y-axis) for individual uninfected chickens (**C**) and “naïve” chickens (**D**) and “vaccinated” chickens (**E**) infected with ER on day 0. Sera were collected on day –3 and on day 15. Trend lines were calculated for each group and sample collection day using the curve fit power mode in the software KaleidaGraph, version 4.1.0 (Synergy Software) and equations and R-values are shown in each panel. For details see “Materials and methods” section.

Results show that on day −3 the mean titer to the ER challenge strain for chickens in the “vaccinated” group had risen slightly compared to pre-vaccination levels but titers showed a large variation between individuals and were not significantly different from those of unvaccinated chickens in the “naïve” and “uninfected” groups (Figure 5A). On day 5 after ER infection titers for chickens in the “vaccinated” group were on average increased more than tenfold and were significantly higher than those of chickens in the two other groups. On day 8 after ER infection titers for chickens in the “naïve” group were also increased and at similar levels as those of chickens in the “vaccinated” group and significantly higher than those of uninfected chickens. After their respective increase, titers for chickens in both infected groups remained at similar levels throughout the experimental period and during this period titers for chickens in the “vaccinated” group were on average higher than those of chickens in the “naïve” group but this difference was not statistically significant.

Comparison of titers to the ER challenge strain with titers to the ER “vaccine related” strain showed that on day –3 titers to the “vaccine related” strain tended to be higher than those to the challenge strain for all three groups of chickens, albeit not statistically significantly so (Figure 5B). At day 15 after infection titers to the “vaccine related” strain had increased for chickens in both groups of infected chickens, but were not statistically significantly higher than at day −3. Titers to the challenge strain were as already mentioned significantly increased on day 15 compared to day −3 for both groups of infected chickens and were in mean higher than those to the “vaccine related” strain on day 15. For vaccinated chickens this difference was statistically significant. Moreover, on the individual level titers were higher to the “vaccine related” strain compared to the challenge strain at sampling on day -3 for all chicken groups (Figures 5C–E). At day 15 however, individual titers were higher to the challenge strain both for “naïve” chickens (Figure 5D) and “vaccinated” chickens (Figure 5E) while the uninfected chickens still showed higher titers to the vaccine related strain (Figure 5C).

Thus, the ER infection elicited clear bacterium specific IgY responses in all infected chickens. For vaccinated chickens this response occurred earlier than for chickens in the “naïve” group. Interestingly, in this experiment vaccination of chickens did not induce significantly increased ER specific IgY in serum collected 10 days after vaccination.

## Discussion

The most striking findings during the current acute phase of infection were the prompt heterophil and MBL responses observed in both naïve and vaccinated chickens. Heterophils are involved in e.g. recognition, phagocytosis and killing of infectious agents as well as the production of cytokines/chemokines that regulate inflammation and ensuing specific immune responses and are hence an important part of innate responses [24, 25]. It thus seems likely that these cells serve a role in the chicken defence against ER infection, both as sentinel cells and regulators of immune responses and as potential effector cells killing ER bacteria. We have indeed shown that when ER was incubated in chicken blood in vitro ER DNA was primarily detected in a leukocyte preparation of blood cultures and not free in plasma and that a significant amount of the bacteria recovered in chicken blood during the acute phase of ER infection were intracellular in leukocytes [20]. Hence, the heterophils may have a role phagocytosing ER in analogy with what has been observed for their mammalian counterparts where phagocytosis and killing of ER in vitro has been shown in porcine and murine neutrophils [14].

Early work indicated the potential role of innate opsonins in the defence against ER infection of naïve hosts. In mice in vivo depletion of complement reduced the time to death after ER infection and pre-treatment of ER with fresh serum reverted this effect and mice even survived the lethal challenge when the bacteria were pre-treated [26]. In the present study we monitored the acute phase protein MBL during ER infection of chickens and found a prompt and substantial increase in serum MBL in response to infection of naïve chickens. MBL is a soluble pattern-recognition receptor belonging to the C-type collecting family that has high affinity for mannose and other carbohydrate residues present on the surface of many infectious agents [27–29]. Chicken MBL seem to function largely as in mammals although some differences have been identified [30, 31]. Regarding bacterial infections, it has been shown that chickens with low base line serum levels of MBL shedded higher numbers of *S. enterica* [32, 33] and were more prone to systemic *Pasteurella multocida* infection [34] upon experimental infection with respective bacterium compared to chickens with high base line MBL levels. A small, approx. 1.3–1.5-fold, transient increase in serum MBL was also observed after *Escherichia coli* infection of chickens [35]. The kinetics of MBL responses during acute infections in chickens have been more closely monitored for infectious bronchitis virus where typically approx twofold increases with peak responses around day 3 after infection were observed [22, 36, 37]. Hence, the serum MBL responses induced by the ER infection in the present study were by far the most pronounced observed in chickens so far. Moreover, the ER bacterium has a polysaccharide capsule with a high content of mannose [38], which is a ligand for MBL [27–29]. Taken together, our results indicate a role for MBL in the chicken immune defence against ER infection e.g. as an innate opsonin.

During the present ER infection we also observed differences in the expression of cell surface receptors CD45, on heterophils and monocytes, and MRC1L-B, on monocytes. For CD45 we observed a decreased expression early after ER infection, day 1 on heterophils and day 3 on monocytes, for cells from chickens in both infected groups, “naïve” and “vaccinated”. CD45 is a transmembrane glycoprotein with intrinsic phosphotyrosine activity that in chickens is expressed on all leukocytes including thrombocytes [39–41]. Chicken CD45 is alternatively spliced to generate at least four distinct isoforms and the monoclonal antibody used in the present study, UM16-6, recognises all four of these [41]. In mammals, the distinct CD45 isoforms are differentially expressed on leukocytes depending on cell type, activation state and differentiation [42, 43] and decreases in expression are predominantly considered a sign of activation [42, 43]. This has not yet been extensively studied in chickens but it has been shown that various T-cell subsets differ in expression of some CD45 isoforms and that activation of T-cells alter their expression of these CD45 isoforms [41]. Thus, the decreased expression of CD45 on heterophils and monocytes during the early ER infection may be due to a down regulation of some or all of the CD45 isoforms, which in turn may reflect an activation of these cell populations. Interestingly, we have observed a similar down regulation of CD45 expression on heterophils upon ER stimulation in vitro (unpublished observation).

On monocytes we observed an initial up regulation, day 1 in “naïve” chickens, followed by a down regulation, days 3 and 5 in both “naïve” and “vaccinated” chickens, of cell surface expression of the chicken mannose receptor MRC1L-B in response to the ER infection. Chicken MRC1L–B is considered a homologue to the mammalian MRC1 [44] that is a carbohydrate-binding endocytic receptor expressed on e.g. populations of macrophages and dendritic cells [45]. The roles of the mannose receptors in the chicken immune response against infectious agents have not yet been elucidated. Nonetheless, an up regulation of MRC1L-B on chicken peripheral blood mononuclear phagocytes after 4 h of in vitro stimulation with cathelicidins, i.e. innate host defences peptides, has been reported [46]. In mammals the MRC1 is considered primarily involved in endocytosis for antigen presentation and not in phagocytosis [45]. Up regulation of MCR1 mRNA expression is considered a marker for so called alternative macrophage activation, M2-like activation, as opposed to proinflammatory and microbicidal M1-activation of macrophages. Hence, one may hypothesise that the altered MRC1L-B expression observed in response to the present ER infection reflects different activation states of the cells or redistribution/recruitment of different monocyte populations in the circulation.

In the present study vaccination of chickens induced a more effective elimination of ER since the recovery of bacteria in blood was clearly reduced compared to that in naïve chickens [20]. Moreover, the acute innate responses in vaccinated chickens were of much shorter duration compared to those in naïve chickens. Vaccination against ER infection has long been practised in both domestic pigs and poultry, mainly turkeys, and although the immune mechanisms of protection have not been clearly elucidated it is generally believed that specific antibody responses constitute at least part of the induced protection [12–14]. Studies in mice have shown that antibodies to ER enhanced both phagocytosis and intra-cellular killing of the bacterium [14]. Pre-infection levels, measured 10 days after the single vaccination, of ER specific IgY were however not significantly different between vaccinated and naïve chickens in the present study. The vaccinated chickens did show significantly increased titers earlier in response to the ER infection than the naïve chickens, day 5 vs. day 8, respectively. Nevertheless, this increase in ER specific IgY occurred after heterophil and MBL responses had returned to baseline levels, i.e. day 3, suggesting that the infection was already eliminated when antibody levels increased. Thus, the role of antibodies in the vaccine induced protection observed here is not clear. The vaccine induced protection may hence involve other mechanisms of the specific immunity. In comparison, in chicken immunity to *Salmonella* that primarily is an intra-macrophage pathogen, T_H_1-type responses and IFN-γ production is considered vital for clearance of the infection [25]. Moreover, a study in bottlenose dolphins (*Tursiops truncates*) showed a correlation between erysipelas vaccine induced IFN-γ production and protection against clinical disease [47].

Taken together, these results give novel insights into chicken immune responses to ER infection and provide a foundation for future more detailed studies of effective defence mechanisms against ER on e.g. the acute phase proteins and phagocytic cells. This infection model did not reflect the dramatic outcome of ER infections often observed in laying hen flocks. Instead our results highlight that even naïve chickens may mount a successful immune defence against this bacterium in the present infection model. This in turn indicates a multifactorial pathogenesis of clinical erysipelas where other factors such as management, stress, co-infection with other infectious agents and overall microbial load, may have a role in the severe outcome of ER infection in the field.

## Supplementary information

**Additional file 1. Experimental outline.** Procedures undertaken in the experimental groups “uninfected” (Uninf), “naïve” and “vaccinated” (Vacc.) with sampling groups A and B, respectively, on the indicated experimental day.

**Additional file 2. Gating strategy for flow cytometry.** Identification of heterophils, monocytes, lymphocytes, thrombocytes and counting beads through singlet gating, FSC/SSC characteristics and using CD45-PerCp/Cy5.5, CD41/61-Fitc and KUL01-PE. From the initial dot-plot in **A** singlet gating (FSC-H vs FSC-A) was performed in **B**. From the singlet gate CD45 high and CD45 intermediate/SSC low events were gated in **C**. From the gate in **C** the SSC high events were gated in **D** and from this gate CD45 high/PE low events were gated as heterophils and CD45 high/PE high events were gated as counting beads in **E**. From the gate in **C** the KUL01 positive events were gated in **F** and from this gate the FSC high events were gated as monocytes in **G**. From the gate in **C** the CD41/61 high/CD45 intermediate events were gated as thrombocytes in **H**. In addition in **H** CD45 high/CD41/61 low were gated and these events were gated on FSC low/SSC low profile as lymphocytes in **I**. A representative chicken blood sample from an uninfected chicken on day –3 is shown. The antibody panel is described in Table 1.

**Additional file 3. Total numbers of thrombocytes in blood.** Chickens were uninfected or experimentally infected with ER on day 0, “naïve” and “vaccinated” groups. Blood samples were collected at the indicated days. Results are shown as group mean values ± 95% CI where non-overlapping CI indicate statistically significant differences. On days –3 and 15 n = 13/group, on days 1, 5 and 8 n = 7/group and on days 3 and 11 n = 6/group. For details see “Materials and methods” section.

## Data Availability

The datasets used and/or analysed during the current study are available from the corresponding author upon reasonable request.

## Abbreviations

BSA: bovine serum albumin
CI: confidence intervals
ER: *Erysipelothrix rhusiopathiae*
MBL: mannose binding lectin
MALDI-TOF MS: matrix-assisted laser desorption/ionization–time-of-flight mass spectrometry
MFI: median fluorescence intensity
PFA: paraformaldehyde
PBS: phosphate buffered saline
