# Supplementary material for: Immune responses upon experimental Erysipelothrix rhusiopathiae infection of naïve and vaccinated chickens
Source: Vet Res. 2020 Sep 14;51:114. doi: 10.1186/s13567-020-00830-9 (PMC7488726; doi:10.1186/s13567-020-00830-9)
Supplement: Supplementary file 1 — Additional file 1. Experimental outline. Procedures undertaken in the experimental groups “uninfected” (Uninf), “naïve” and “vaccinated” (Vacc.) with sampling groups A and B, respectively, on the indicated experimental day. [file 13567_2020_830_MOESM1_ESM.pdf]

Additional file 1: Table S1. Experimental outline with procedures undertaken in the experimental groups “uninfected” (Uninf), “naïve” and “vaccinated” (Vacc.) with sampling groups A and B, respectively, on the indicated experimental days

		**Experimental day**																		
		-18	***-13***	***-3***	***0***	***1***	***2***	***3***	***4***	***5***	***6***	***7***	***8***	***9***	***10***	***11***	***12***	***13***	***14***	***15***
**Uninf.**	**A** *n=7*	W	BW	BW		BW	W	W	W	BW			W	W	W	BW	W			BWP
n=13	**B** *n=6*	W	BW	BW		W	W	BW	W	W			BW	W	W	W	W			BWP
**Naïve**	**A** *n=7*	W	BW	BW	I	BW	W	W	W	BW			W	W	W	BW	W			BWP
n=13	**B** *n=6*	W	BW	BW	I	W	W	BW	W	W			BW	W	W	W	W			BWP
**Vacc.**	**A** *n=7*	W	VBW	BW	I	BW	W	W	W	BW			W	W	W	BW	W			BWP
n=13	**B** *n=6*	W	VBW	BW	I	W	W	BW	W	W			BW	W	W	W	W			BWP

W – weighing
V – vaccination
B – blood sampling
I – infection
P – *Post mortem* examination
