# Supplementary figures and images for: Immune responses upon experimental Erysipelothrix rhusiopathiae infection of naïve and vaccinated chickens
Source: Vet Res. 2020 Sep 14;51:114. doi: 10.1186/s13567-020-00830-9 (PMC7488726; doi:10.1186/s13567-020-00830-9)

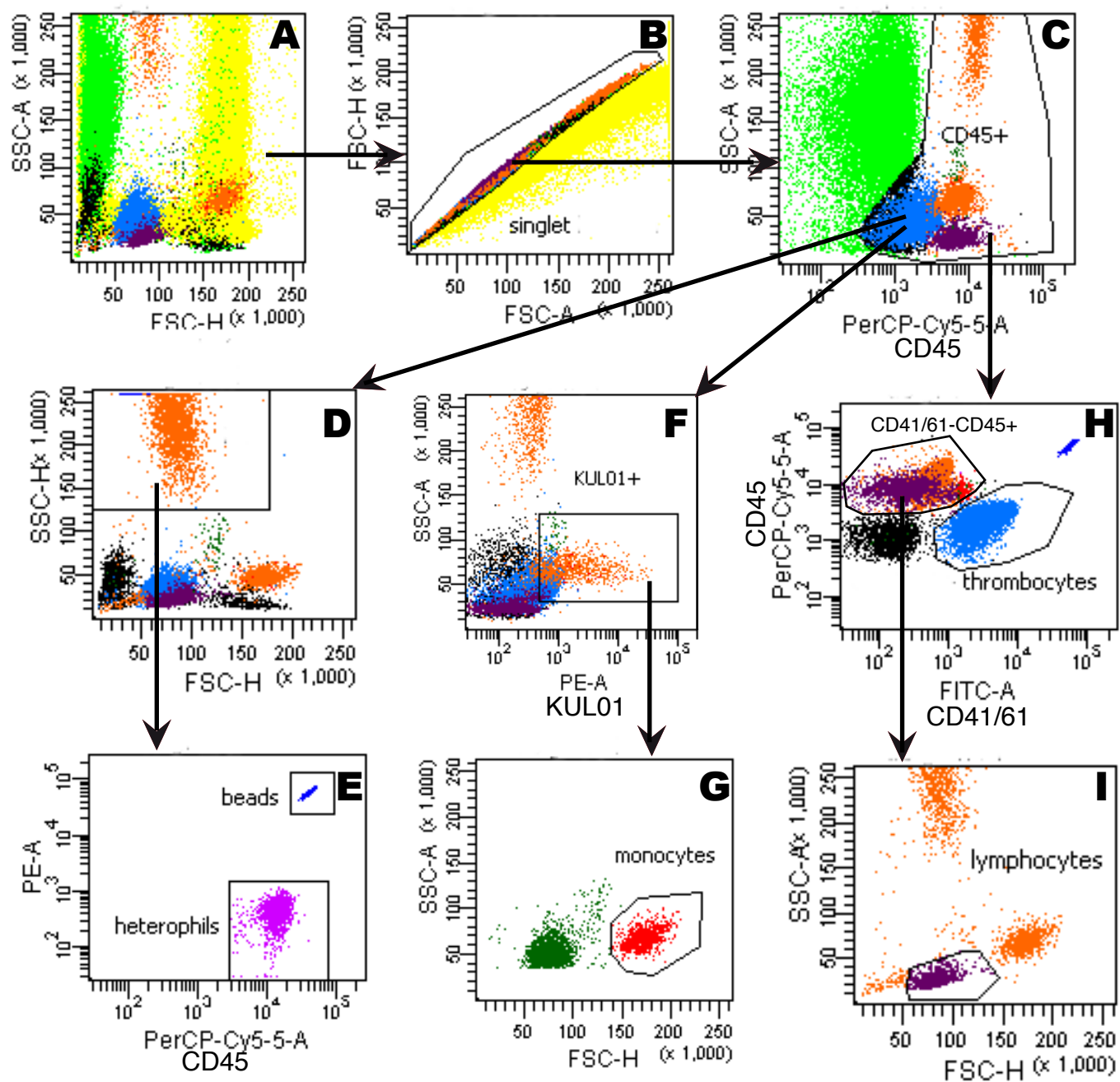

Supplement: Supplementary file 2 — Additional file 2. Gating strategy for flow cytometry. Identification of heterophils, monocytes, lymphocytes, thrombocytes and counting beads through singlet gating, FSC/SSC characteristics and using CD45-PerCp/Cy5.5, CD41/61-Fitc and KUL01-PE. From the initial dot-plot in A singlet gating (FSC-H vs FSC-A) was performed in B. From the singlet gate CD45 high and CD45 intermediate/SSC low events were gated in C. From the gate in C the SSC high events were gated in D and from this gate CD45 high/PE low events were gated as heterophils and CD45 high/PE high events were gated as counting beads in E. From the gate in C the KUL01 positive events were gated in F and from this gate the FSC high events were gated as monocytes in G. From the gate in C the CD41/61 high/CD45 intermediate events were gated as thrombocytes in H. In addition in H CD45 high/CD41/61 low were gated and these events were gated on FSC low/SSC low profile as lymphocytes in I. A representative chicken blood sample from an uninfected chicken on day –3 is shown. The antibody panel is described in Table 1. [file 13567_2020_830_MOESM2_ESM.pdf]

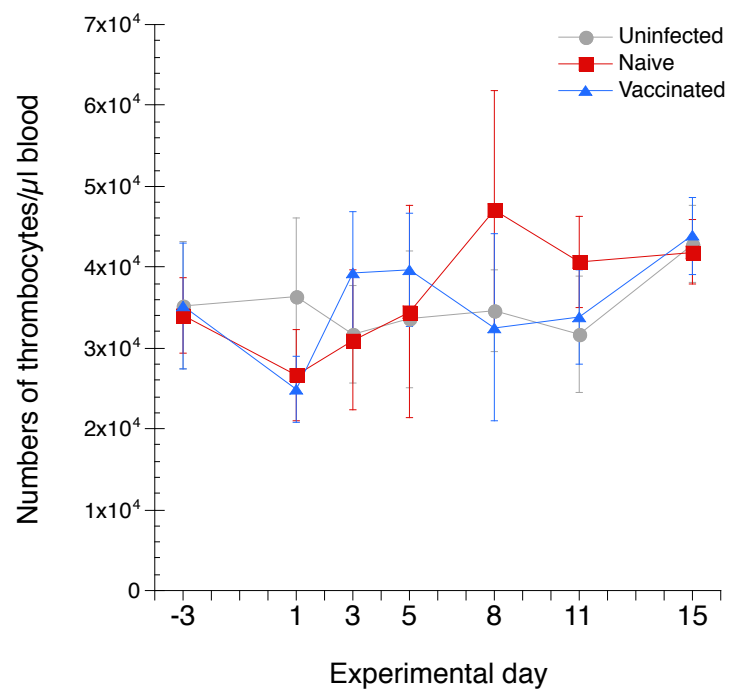

Supplement: Supplementary file 3 — Additional file 3. Total numbers of thrombocytes in blood. Chickens were uninfected or experimentally infected with ER on day 0, “naïve” and “vaccinated” groups. Blood samples were collected at the indicated days. Results are shown as group mean values ± 95% CI where non-overlapping CI indicate statistically significant differences. On days –3 and 15 n = 13/group, on days 1, 5 and 8 n = 7/group and on days 3 and 11 n = 6/group. For details see “Materials and methods” section. [file 13567_2020_830_MOESM3_ESM.pdf]
